# Perceived Barriers, Facilitators, and Needs Related to Promoting Physical Activity in Cancer Care: Qualitative Insights from Oncology Care Providers

**DOI:** 10.3390/cancers17142281

**Published:** 2025-07-09

**Authors:** Gaurav Kumar, Priyanka Chaudhary, Apar Kishor Ganti, Jungyoon Kim, Lynette M. Smith, Dejun Su

**Affiliations:** 1 TSET Health Promotion Research Center, Stephenson Cancer Center, The University of Oklahoma Health Sciences, Oklahoma City, OK 73104, USA; 2 Pennington Biomedical Research Center, Baton Rouge, LA 70808, USA; priyanka.chaudhary@pbrc.edu; 3 Internal Medicine Division of Oncology & Hematology, University of Nebraska Medical Center, Omaha, NE 68198, USA; aganti@unmc.edu; 4 VA Nebraska Western Iowa Health Care System, Omaha, NE 68105, USA; 5 Department of Health Services Research & Administration, College of Public Health, University of Nebraska Medical Center, Omaha, NE 68198, USA; jungyoon.kim@unmc.edu; 6 Department of Biostatistics, College of Public Health, University of Nebraska Medical Center, Omaha, NE 68198, USA; lmsmith@unmc.edu; 7 Department of Health Promotion, College of Public Health, University of Nebraska Medical Center, Omaha, NE 68198, USA; dejun.su@unmc.edu

**Keywords:** oncology care providers, barriers, facilitators, physical activity, qualitative study

## Abstract

**Background:** Physical activity (PA) is associated with lower mortality and cancer recurrence risks. Although evidence shows health benefits for cancer patients before, during, and immediately after treatment, PA recommendations are not regularly included in the standard care. **Objective:** The study aimed to identify perceived knowledge, barriers, and facilitators of oncology providers’ PA promotion for cancer patients using the 5A (Assess, Advise, Agree, Assist, and Arrange) framework. **Methods:** A qualitative research design with a phenomenological approach was adopted. A purposive sample of 16 oncology care providers in Nebraska participated in semi-structured interviews via Zoom/phone. Interviews were audio-recorded, transcribed, and imported into MAXQDA 2024 for thematic analysis. **Results:** Analysis of the qualitative data identified five themes: (i) Broad and inclusive conceptualizations of PA among oncology care providers suggested that they were able to define PA; (ii) Current Practices in PA Counseling included advising on PA and assessment; (iii) Barriers to PA counseling included lack of guideline awareness, insufficient training, low prioritization, uncertainty about responsibility, time constraints, limited resources, lack of referral systems, patient health conditions, and environmental factors; (iv) Facilitators were identified as acknowledged health benefits for cancer survivors, awareness of PA recommendations, access to community resources, and support from interdisciplinary teams; and (v) Expressed desire among oncology care providers for training on incorporating PA into oncology care. **Conclusions:** Oncology providers recognized PA’s health benefits for cancer survivors but did not promote it due to inadequate knowledge of guidelines and lack of resources. These barriers require improved PA counselling education to help providers incorporate PA into clinical care.

## Simple Summary

Regular physical activity can help people with cancer recover and feel better, but oncology care providers (e.g., oncologists, nurses, and allied health professionals) often struggle to include physical activity advice in routine care. We interviewed 16 cancer doctors, nurses, and support staff to learn how they view physical activity counseling, the obstacles they face, and what would help them support cancer survivors in staying active. While providers value physical activity, they reported barriers such as limited training, unclear guidelines, time constraints, and patient health concerns. To address these gaps, we recommend improving provider education, offering practical exercise guidelines, fostering interdisciplinary collaboration, and implementing supportive policies and reimbursement models. Future research should explore scalable strategies to promote physical activity in oncology care. These efforts can enhance survivorship care and improve long-term health outcomes.

## 1. Introduction

Advancements in early detection and treatment have markedly enhanced cancer survival rates, with 69% of patients surviving beyond five years post-diagnosis [1]. Despite this positive trend, cancer treatments such as surgery, hormone therapy, radiation therapy, and chemotherapy can induce immediate, late, and long-term side effects such as cancer-related fatigue, musculoskeletal pain, metabolic dysfunction, cardiovascular impairment, cognitive difficulties, and mental health challenges [2,3,4,5,6,7,8,9]. These effects often lead to reduced quality of life and pose significant challenges to optimal survivorship outcomes [10,11,12].

Research has consistently indicated that regular physical activity (PA) can alleviate many adverse side effects associated with cancer treatments [13,14]. Specifically, PA has been demonstrated to significantly enhance muscular strength, aerobic endurance, physical function, and various aspects of quality of life (QOL), including improved sexual health and reduced fatigue [15,16,17,18,19,20,21]. Furthermore, there was evidence that PA may decrease the likelihood of developing other chronic diseases (e.g., cardiovascular disease or hypertension) and confer survival benefits across diverse groups of cancer patients [22,23,24]. The American Cancer Society and the American College of Sports Medicine have issued PA guidelines for cancer survivors that advocate for 150–300 min per week of moderate-intensity or 75–150 min per week of vigorous-intensity PA, along with muscle-strengthening activities on two or more days per week tailored to the specific needs of each cancer survivor [25,26].

Despite the established health benefits of PA and the issuance of PA guidelines, a notable disparity persists in PA participation among cancer patients when compared to healthy individuals of similar ages, with many cancer patients failing to engage in sufficient PA to recoup related health benefits [27,28]. Research indicates that most cancer survivors are inadequately active, with adherence to PA guidelines ranging from 17% to 47% [29,30,31]. It thus becomes imperative to identify effective strategies to inform and motivate cancer patients to engage in PA.

Although cancer patients express a desire for PA guidance, they frequently do not receive advice or counseling from their oncology care providers (e.g., oncologists and oncology nurses), who are their preferred sources of information [32,33,34]. Less than 40% of cancer patients reported receiving PA recommendations from oncologists during and after treatment [35,36,37]. Consequently, it has been recommended that a multidisciplinary team of oncology care providers, including oncologists, oncology nurses, oncology nurse practitioners, patient navigators, and oncology social workers, should collectively assume the responsibility of discussing PA recommendations with cancer survivors to enhance their PA levels [38,39].

Barriers and facilitators influencing oncology care providers’ routine provision of PA recommendations to cancer patients remain under investigation. A comprehensive understanding of oncology care providers’ strategies for promoting PA and the challenges associated with such promotion can inform future interventions and potentially influence their training in the cancer care continuum. Therefore, this study aimed to explore oncology care providers’ perceptions, practices, and preferences regarding PA counseling, with a focus on identifying key barriers and facilitators within oncology care settings.

## 2. Methods

### 2.1. Study Design

A qualitative phenomenological approach was employed to explore the roles, opinions, experiences, and recommendations of oncology care providers with regard to the provision of PA counseling for cancer survivors [40]. This approach focuses on examining phenomena by illuminating experiences and understanding the meaning attributed to participants [41]. It is grounded in the philosophy of social constructivism, emphasizing the interpretation of events and life through a sociocultural and historical perspective. This involves an iterative process to gain insight into participants’ viewpoints, thereby constructing a more comprehensive understanding of their lived experiences [42].

### 2.2. Sampling and Recruitment

Eligibility for participation was restricted to individuals providing direct care and treatment to patients with cancer. A purposive sampling method was employed to recruit 16 oncology care providers (4 male and 12 female) from diverse demographic groups within a large hospital in the Midwest region. Oncology care providers include physicians (surgical oncologists, medical oncologists, and radiation oncologists), nurses (advanced practice nurses and registered nurses), and allied health professionals (physician assistants, dieticians, and oncology social workers). The recruitment target was to enlist 16 oncology care providers. Previous research indicates that 8 to 16 in depth interviews can achieve 80–90% data saturation [43,44].

A total of 16 participants were recruited through purposive and snowball sampling [43]. Recruitment was conducted by disseminating digital flyers to community partners (oncology clinics and hospitals) through social media, email, and referrals from other potential participants. The flyer provided a brief introduction to the study, the eligibility criteria, and the contact information. Eligible participants were contacted by the lead author (GK) using their preferred method of communication (i.e., text, email, or phone call) to schedule an interview at a time and date convenient for them.

### 2.3. Data Collection

Data were collected from August 2023 to February 2024 through semi-structured interviews via Zoom and/or telephone, based on participants’ preferences. The interview guide was developed to elicit oncology care providers’ perceptions and practices regarding PA counseling, including barriers and facilitators. During the interview introduction, participants were informed that the term “cancer survivor” referred to individuals from the point of diagnosis through the remainder of life. The interviews were conducted by the lead author, who has significant qualitative interview experience and was trained by a faculty mentor (DS) with over 15 years of research experience. The interviewer maintained a neutral stance throughout all interviews to reduce bias and avoid leading questions. A “think aloud” cognitive interview approach was used to assess face validity and determine interview duration [45]. The guide was pilot tested by an oncological care provider from Nebraska Medicine before being finalized for use in the data collection. Verbal informed consent was obtained before each interview. Semi-structured interviews were conducted by one investigator (GK), with additional probing questions used when needed for clarity or detail. Each interview lasted approximately 25 min and was audio recorded with permission. After the interview, the interviewer made field notes regarding mood, gestures, and noteworthy occurrences (gestures were not applicable to telephone interviews). The participants received a USD 50 prepaid Visa gift card as compensation for participation in the study.

### 2.4. Semi-Structured Interview

The interview guide was developed in accordance with the 5A guidelines (Document S1 in Supplementary Materials), which involved clinicians inquiring about (or assessing), advising, agreeing upon, assisting with, and arranging follow-up concerning patients’ efforts to change their behavior. This framework serves as a model for brief counseling that can encourage PA [46]. It is employed to discuss current behavior, recommend changes, evaluate readiness for change, assist with goal setting, and schedule follow-up. The 5As have been validated as a cohesive framework for behavioral therapy in primary care [46]. Consequently, to ensure the objectivity and trustworthiness of our study, we developed a qualitative semi-structured interview guide based on the 5As framework. This approach enhanced the credibility of our findings, rendering them more reliable and credible [47].

### 2.5. Ethical Considerations

This study was approved by the University of Nebraska Medical Center Institutional Review Board (IRB# 0468-23-EX). The study objectives and voluntary participation were explained to the participants, and informed consent was obtained before beginning semi-structured interviews. Confidentiality was assured by using a code number instead of names (e.g., staff physician, registered nurse, physician assistant, etc.) and removing identifying information such as their name and affiliation from the transcripts before data analysis. All audio recordings and transcripts were saved securely. The Standards for Reporting Qualitative Research (SRQR) guidelines were followed in this study [48].

### 2.6. Data Analysis

The interviews with oncology care providers were audio-recorded, transcribed verbatim, and anonymized. Initial automatic transcription was performed using Otter.ai©, followed by meticulous review and correction by the lead author to ensure accuracy. The transcripts were subsequently uploaded to MAXQDA 2024 (VERBI Software, 2024) for data analysis.

The study utilized thematic analysis to demonstrate the concepts of framework analysis following the five processes of familiarization: defining a thematic framework, indexing, charting, mapping, and interpretation [49]. To ensure data saturation, preliminary analysis was conducted concurrently with data collection. After coding and reviewing the 14th transcript, no new themes emerged, suggesting thematic saturation had been reached. The final two interviews confirmed consistency with existing themes, indicating that further data collection was unnecessary. The analysis involved reading transcriptions multiple times to understand participants and their responses, identifying important phrases and rephrasing them broadly, forming and confirming meanings through team discussions, categorizing themes, and creating a comprehensive description of themes.

The rigor of the study was achieved by employing well-known qualitative research methodologies to enhance reliability and dependability. Peer debriefing and triangulation methods were employed to examine participants’ responses and produce qualitative thematic results [43]. The lead author deductively created a codebook using semi-structured interview questions. Two researchers, GK and PC, reviewed five randomly chosen transcripts representing approximately 25% of all the participants and coded them according to the codebook. The coding was checked for similarities and inconsistencies, and both researchers met to discuss any conflicts in the coding. The lead author continued coding the transcripts because of the high level of agreement. The researchers took notes during the coding process to discover similarities in oncology care providers’ responses to the interview questions and preliminarily identify relevant themes [50].

The lead author examined the coded transcripts and notes from the coding process to develop and enhance themes in the data. The lead author (GK) aimed to identify three to five main themes in the responses of oncology care providers [51]. Themes and subthemes were discussed with the research team (PC) so that they could be assigned more succinct titles and meanings. The two researchers then conducted a final review of all coded data and met to resolve any discrepancies through discussion until full consensus was achieved.

## 3. Results

### 3.1. Sample Characteristics

In total, 16 oncology care providers completed the semi-structured interviews. Participants were predominantly middle-aged, with 43.8% aged between 40 and 49 years and a female majority (75%). The racial and ethnic breakdown of the participants showed that 75% were white, while Hispanics and Asians each accounted for 12.5%. Most participants were married (87.5%) and had worked in various clinical specialties, with 50% being staff physicians. The most common practice area was medical oncology (52.2%), which treated mixed cancer cases (37.5%). Most participants (93.3%) provided outpatient care, and the average number of years of practice was 11.75 years. For additional sociodemographic information, see Table 1.

### 3.2. Qualitative Findings

The qualitative data findings identified five emerging themes: (i) Broad and inclusive conceptualizations of PA among oncology care providers; (ii) current practices in PA counseling by oncology care providers; (iii) barriers faced by oncology care providers to PA counseling; (iv) facilitators for PA counseling; and (iv) desire expressed by oncology care providers for PA training and education (Table 2).

#### 3.2.1. Theme 1: Broad and Inclusive Conceptualizations of Physical Activity Among Oncology Care Providers

This theme highlights a comprehensive and inclusive understanding of what constitutes PA in the context of oncology care. Oncology care providers broadly define PA as any form of movement that breaks the cycle of sedentariness, extending beyond traditional exercises to include daily tasks and movements. A participant illustrates the diverse nature of PA by referencing *“being active in just about any capacity, whether it’s walking, aerobics, yoga” [Nurse Practitioner].* Another oncology care provider illustrates a broad sense of PA *“Physical activity to me is very broad. I would say all like being physically active, not being sedentary, and moving around” [Staff Physician].* Other oncology care providers have discussed the incorporation of both structured and everyday activities.

#### 3.2.2. Theme 2: Current Practices in PA Counseling by Oncology Care Providers

Related to current practice in PA counseling, two subthemes were identified: advice and assessment of PA.

##### Advice on Physical Activity

The subtheme of PA advice from oncology care providers reveals the provision of tailored advice with small and achievable goals, which is a supportive approach for encouraging PA among cancer patients. Encouraging small, achievable goals is a common strategy to build confidence and motivation; for instance, one stated, *“Usually, we start with small goals … to make them doable, manageable and seem achievable” [Staff Physician].* This approach helps patients gain trust in their bodies and the process of reintegrating PA into their lives.

The advice is inherently flexible, acknowledging that the amount and type of activity will vary greatly among cancer survivors: *“It’s pretty much whatever they’re able to do. So, be very individualized” [Staff Physician].* This flexibility allows for adjustments based on the patient’s health status and response to treatment. Oncology care providers often start with a foundational goal that is personalized, considering the patient’s pre-treatment activities, physical limitations, and personal preferences.

##### Assessment of Physical Activity

The assessment of PA by oncology care providers encompasses various strategies for understanding and gauging PA levels in cancer patients. Most oncology care providers verbally assessed their patients’ PA levels. Oncology care providers gather insights into patients’ social and previous histories, often during initial screenings, to identify past and current PA engagement. One provider explained, *“Well, when they’re first coming in for screening, we ask a lot of their social and previous history where they’ll normally say like, I’m an avid runner, or I used to play softball” [Registered Nurse].* This initial assessment helps to understand the patients’ baseline and potential interest in physical activities. Conversations about PA extend beyond identifying activities. Oncology care providers also delve into the details of patients’ daily routines to understand their mobility and sedentary patterns. The comprehensive inquiry covers various aspects, from job-related activities to leisure time, and provides a holistic view of the patient’s lifestyle.

Additionally, to systematically evaluate PA needs, specific tools and guidelines are employed, such as the Short Physical Performance Battery (SPPB) and the National Comprehensive Cancer Network (NCCN) guidelines, which include questions tailored to cancer survivors: *“We do a short performance physical battery with our bone marrow transplant survivors and kind of see where they are at with balance and walking, kind of make our recommendations from there” [Nurse Practitioner]* The approach to assessing PA among cancer patients by oncology care providers is characterized by a detailed, patient-centered methodology. Specific assessment tools were used through personalized inquiries.

#### 3.2.3. Theme 3: Barriers Related to Physical ACTIVITY Counseling

##### Lack of Knowledge Related to Physical Activity Guidelines

This subtheme reflects oncology care providers’ acknowledgment of a gap in specific, universally accepted PA guidelines for cancer patients. Oncology care providers expressed uncertainty about the existence of detailed PA guidelines, indicating a significant barrier to offering consistent PA counseling or advice. For example, one provider stated, *“I don’t know if there is a specific guideline” [Physician Assistant],* highlighting the ambiguity surrounding the PA recommendations. Another remarked, “*I’m not really aware of any particular guideline-based recommendations” [Staff Physician],* underscoring the perceived absence of clear, authoritative PA guidelines for cancer survivors. The responses further highlight a profound uncertainty about guidelines; for example, *“I think that there is some recommendations from the American Cancer Society and Susan Komen … but I don’t know what they are actually like and the exact specifications of them” [Nurse Practitioner].* This shortfall is not confined to a lack of awareness about general guidelines; it extends to profound uncertainty about any specific guidelines tailored to the needs of individual cancer patients or survivors.

##### Lack of Training Related to Physical Activity Counseling

Oncology care providers revealed a significant barrier in their practice: a pronounced deficiency in formal training and education related to counseling cancer patients on PA. The gap is highlighted by oncology care providers’ observation that education/training in PA indicates a systematic shortfall in healthcare education. For example, the oncology care providers echoed that *“wasn’t ever touched [PA] upon with any population, really, but definitely not cancer patients” [Staff Physician].* The brevity of this training is further lamented by other providers, who describe their education on PA for cancer patients as *“minimal and likely very vague” [Nurse Practitioner]* and *“Very little, probably” [Staff Physician].* Such statements underscore the inadequacy of current training programs to equip healthcare providers with the knowledge necessary to offer patient-specific PA advice.

##### Low-Priority for Physical Activity Counseling

This subtheme reflects a widespread lack of emphasis on PA counseling for cancer survivors among oncology care providers. A notable response is acknowledging a lower priority, for example, *“I don’t ask specific questions on their current physical levels of physical activity” [Staff Physician].* Furthermore, cultural reflections such as *“it’s not in our culture to be active” [Nurse Practitioner]* and direct admissions of not asking about patients’ PA levels underscore broader societal and clinical oversight.

##### Not Being the Right Person to Advise on Physical Activity

When asked about current practices in PA counseling, oncology care providers indicated that they felt unqualified or inappropriate to provide PA counseling. Oncology care providers suggest reasons such as not being part of the patient’s long-term care team or feeling that their primary role does not include prescribing PA. For example, *“it’s not part of a checklist … I see a patient maybe sometimes one or two years out from surgery, and I don’t really address physical activity” [Staff Physician]* illustrates a common belief that their position does not align with providing PA counseling. Additionally, the oncology care provider acknowledges the potential value of PA but recognizes their role’s limitations, which articulates this cautious approach: “*Sometimes professionally, it may not be my place to talk about physical activity*” *[Staff Physician]*.

##### Time Constraints Due to Other Clinical Responsibilities

This subtheme points to the barriers oncology care providers face due to time constraints, which hampers their ability to offer comprehensive counseling on PA to cancer patients. Oncology care providers articulate the challenges of limited appointment durations, noting, *“the physicians don’t seem to have time in their clinical role” [Physician Assistant],* and highlighting the struggle for those with high caseloads, *“But if you were to ask like a case manager with a higher load, they wouldn’t have enough time” [Social Worker].* Another provider vividly describes the effects of time constraints in clinical practice, detailing the challenge of addressing a patient’s comprehensive needs within a limited timeframe. For example, *“a new patient, you get an hour … Follow-up patients get 20 min” [Staff Physician].* Furthermore, it emphasizes the brevity of patient interactions, highlighting the prioritization of immediate medical concerns over wellness and lifestyle advice such as PA.

##### Limited Resources for Physical Activity Counseling

This subtheme underscores the limited knowledge of accessible resources in the community. The availability or lack of awareness of supportive materials or programs, such as *“Honestly, nothing. Because we don’t have any particular thing that we tell patients … We really don’t have any resources that we can provide to the patient” [Registered Nurse],* pointing to the absence of tangible support for patient engagement in PA. Further, the disconnect between potential referral sources or past programs and current practice is touched upon, with comments like *“I think there used to be like a LIVESTRONG for Survivor. I don’t even know if that’s around anymore” [Nurse Practitioner].*

##### Limited Referral Opportunities

This theme reflects a gap in oncology care, where there is a noticeable deficiency in directing patients towards physical therapy (PT) or occupational therapy (OT) for PA advice. One participant noted the disparity in patient care, stating, *“our physicians, I’m so sorry. I don’t see our physicians referring them to PT or OT either” [Registered Nurse],* another highlighted the procedural barriers with the following: *“But I don’t refer unless the doctor tells me to”-Registered Nurse]*, reflecting a systemic reliance on physician directives that can hinder timely and proactive referrals, ultimately limiting support for patients seeking PA-related services.

##### Inadequate Health Status of Cancer Survivors

Limited health conditions as a barrier to PA counseling in oncology care emerge from the insights shared by oncology care providers. These limitations encompass a wide range of physical, mental, and emotional challenges faced by cancer patients, which significantly impact their ability to engage in PA. One participant notes the direct impact of patients’ current health status on PA counseling, stating, *“And obviously, if it’s going to hurt them, if they’re already losing weight and not eating enough, I’m not going to tell them to increase physical activity” [Staff Physician]*.

Specific health conditions such as neuropathy, fatigue, and the effects of surgery or chemotherapy introduce additional complications. For instance, *“Oh, just long-term side effects from their treatments make it difficult if they have neuropathy. Fatigue? I think those are the big ones” [Staff Physician]*. These quotes underline diverse health issues that can deter PA engagement. Additionally, the emotional and mental toll of cancer treatment, including depression and loss of motivation, further complicates patients’ ability to engage in PA.

##### Environmental Issues

This subtheme highlights how patients’ living environments can significantly hinder their ability to engage in physical activity (PA). Oncology care providers noted that perceived neighborhood safety and inadequate infrastructure often discourage outdoor PA. One participant shared, “*I have patients that live in different parts of Omaha that don’t feel safe walking outside” [Social Worker]* emphasizing how safety concerns can serve as a powerful deterrent. Physical barriers such as poor sidewalk conditions or the complete absence of sidewalks further compound the issue, particularly in older parts of town.

The risk of infection is another significant environmental barrier for cancer patients, whose immune systems are often compromised by their treatment. As one provider explained, *“some of them have to be very aware of their outdoor settings as far as risk for an infection, depending on air contaminants” [Staff Physician]*, highlighting the need for patients to navigate their environments carefully to avoid potential health risks. Additionally, the current global health landscape further complicates this, as pointed out by one oncology care provider, *“ They [cancer survivors] have to be careful about getting in contact with patients who are or people who are sick, changing of the seasons, you get the flu. Now we have COVID” [Staff Physician]*, which highlights the increased vulnerability of cancer patients to infectious diseases and the need for careful consideration of when and where to engage in PA.

#### 3.2.4. Theme 4: Facilitators of Physical Activity Counseling

##### Health Benefits Related to Physical Activity

The health benefits of PA for cancer patients are widely recognized and emphasized by oncology care providers, highlighting its significant impact on both physical and mental health. They highlight how PA enhances circulation, lung health, and cardiovascular fitness and significantly benefits mental health. For instance, *“Definitely mental health … I mean obviously also improves your circulation, lung health, cardiovascular” [Staff Physician]*.

Moreover, oncology care providers acknowledge that PA protects against cancer risks and recurrence. For example, *“I know that obesity is associated with cancer risk and also cancer recurrence for several different cancer types and that PA or being physically active is protective for cancer risk, even irrespective of weight” [Registered Dietician]*. PA was also credited with alleviating treatment-related side effects, including fatigue and chemotherapy-induced peripheral neuropathy, while enhancing energy levels, physical strength, and overall recovery.

##### Knowledge of Oncology Care Providers on Physical Activity Guidelines

Oncology care providers play a crucial role in promoting PA among cancer survivors by leveraging established guidelines from reputable organizations. One provider recommends *“150 min of moderate-intensity physical activity per week for cancer survivors” [Nurse Practitioner],* aligning with widely recognized standards for maintaining health and supporting recovery. This recommendation is echoed by the American Institute for Cancer Research, which, as another provider notes, suggests *“75 min of high intensity, physical activity”* alongside *“stretching and weight training, two days a week, at least two days a week” [Staff Physician].* References to the American Cancer Society’s (ACS) PA guidelines further reinforce the consistency of these recommendations across professional sources, with another provider stating, *“Yes, we commonly reference American Cancer Society physical activity guidelines” [Nurse Practitioner].* These guidelines offer a structured approach to PA that balances intensity, frequency, and variety to support the overall health and recovery of cancer survivors.

##### Resources Available in the Community

The availability of community resources to support PA among cancer survivors enables a proactive approach by oncology care providers to integrate PA into survivorship care. Insights from oncology care providers reveal initiatives such as clinical trials offering health coaching and Fitbits, yoga programs, and specific PA-focused programs in the YMCA.

One innovative example involved a clinical trial where participants were provided with Fitbits and received personalized support: “*A clinical trial where they are looking at so they give patients a Fitbit, and they get health coaching” [Staff Physician].* highlighting how digital tools and coaching can foster sustained PA engagement. Additionally, gentle and accessible options like yoga were mentioned as beneficial for survivors managing fatigue or mobility issues. One provider shared, *“A yoga program at the Village Pointe campus that they do have about once a week and may be offered for free to one patient a month” [Nurse Practitioner].* illustrating community-based support tailored to survivors’ needs.

The LIVESTRONG program at the YMCA was frequently cited as a key resource, offering structured PA programming designed specifically for cancer survivors. As one participant noted, *“There is a program, the LIVESTRONG program … that helps quite a bit” [Nurse Practitioner].* Other community resources mentioned included “*A Time to Heal … Project Pink” [Social Worker]* and even creative at-home solutions like the *“chicken soup workout” [Registered Nurse]* on YouTube. Collectively, these programs exemplify the diverse, accessible, and supportive PA options available to cancer survivors through community engagement. By leveraging technology, specialized programs, and accessible fitness opportunities, the providers aim to facilitate PA as a key component of cancer recovery and long-term health.

##### Interdisciplinary Support

The collective insights from oncology care providers emphasize a team-based approach to oncology care, where the encouragement and facilitation of PA are viewed as a shared responsibility.

Interdisciplinary referral processes have emerged as another facilitator in the holistic care of cancer patients, underscoring the collaborative effort among oncology care providers to integrate PA into patient care. These referrals span a range of specialties, including psychiatry, PT, OT, nutrition, and lymphedema therapy, highlighting the diverse needs of cancer survivors and providing a comprehensive approach to addressing these needs. One provider noted the importance of addressing mental health alongside physical health, stating, *“If we notice that our patients have any troubles with depression, we do recommend them to our psychiatrist. And I know they recommend activity” [Registered Nurse].* This emphasizes the recognition of the interplay between physical and mental well-being and the role of PA in improving mental health. Frequent referral to PT is highlighted by several providers, reflecting the critical role of physical therapy in cancer recovery: *“I refer to PT a lot … I refer them to physical therapy, a lot of times, many of the lymphedema from breast surgery “ [Physician Assistant].* These referrals are tailored to address specific physical challenges faced by cancer survivors, such as lymphedema, muscle aches, and reduced range of motion. Additionally, the role of nurse navigators in facilitating these referrals is also highlighted, indicating an infrastructure that supports efficient coordination of care.

Support from other interdisciplinary providers also facilitates PA counseling for cancer patients, underscoring a collaborative, multidisciplinary approach within oncology care. This comprehensive support network involves a diverse range of professionals, including case managers, fellows, surgeons, radiation oncologists, medical oncologists, nurse case managers, physical therapists, and advanced survivorship practice providers, all of whom play a vital role in discussing and encouraging PA as part of patient care. One provider explained the integrated approach within their clinic, stating, *“my case manager discusses [PA] with patient. Then I have fellows in my clinic, they also discuss [PA] with patients, surgeons, radiation oncologists who are on our team” [Staff Physician].* Additionally, nurse case managers and survivorship advanced practice providers are specifically mentioned for their roles in discussing PA with patients. This indicates that, beyond immediate medical care, there is an infrastructure to support patients in adopting and maintaining PA as part of their lifestyle post-treatment.

#### 3.2.5. Theme 5: Desire for Physical Activity Training and Education

##### More Education on Physical Activity

Oncology care providers have expressed keen interest in expanding their knowledge and resources regarding PA to enhance their support for cancer survivors. One foundational aspect the provider mentioned was understanding the patient’s ability to perform daily activities, as highlighted by the statement, *“I want to know if they’re able to do their activities of daily living and unable to function” [Nurse Practitioner].* This concern underlines the importance of assessing patients’ functional capacity. The need for more information about the available resources for PA has also emerged. Providers are looking for *“information on [PA] resources that maybe I don’t know about would be” [Staff Physician],* indicating a desire to discover new avenues to support patients’ engagement in PA. Similarly, for the sentiment, *“I would say that some just being directed to the resources can be helpful” [Registered Dietician], which* suggests that accessing well-curated information on PA resources could greatly enhance providers’ ability to guide their patients. Moreover, staying updated with the latest PA guidelines is another area where providers see room for improvement, as one mentions, *“I need to get up to date on my [PA] guidelines” [Staff Physician].* This reflects a commitment to ensuring that patient advice is grounded in current evidence and recommendations. Lastly, the value placed on patient educational materials is evident, with providers seeking *“very practical resources, infographic type stuff that I can use for patients” [Staff Physician].* Such materials are crucial for simplifying complex information and making it more digestible and actionable for patients.

##### Training

Oncology care providers preferred virtual and online training due to its convenience and flexibility. Virtual training methods, such as webinars and Zoom meetings, are highlighted for their accessibility and ease of integration into providers’ schedules. One provider noted, “*I feel like it’s easier to have webinars or, Zoom. Meeting conferences are too fine, but many people if it is more accessible if we do virtual” [Staff Physician]*, emphasizing the appeal of virtual learning environments in today’s healthcare setting.

However, the importance of in-person training for immersive learning experiences and opportunities for direct interaction is also recognized. Despite the widespread preference for virtual training, the value of in-person learning experiences is also acknowledged, particularly for the hands-on engagement and direct feedback they offer: *“I like in person … I learned better when I see someone do something and then they can critique” [Registered Nurse].* These perspectives highlight the perceived benefits of traditional learning environments for fostering deeper engagement and understanding.

## 4. Discussion

Increasing evidence supports the assessment of PA as a vital sign among cancer survivors by oncology care providers [14,39,52]. Assessing patients’ PA during medical appointments might serve as a reminder to the patient, regardless of previous adherence, to engage in PA [53,54]. Inquiring about patients’ PA behavior indicates that their oncology care providers value the significance of PA in their well-being and recovery [55,56].

Findings from the study offer a unique perspective from oncology care providers on the concept of PA, presenting a broader interpretation that transcends the conventional view of cancer care. This research is among the first to explore how oncology care providers perceive ‘physical activity,’ which they define not merely as scheduled exercise routines but as any form of movement that counters sedentary behavior. This inclusive understanding of PA extends prior work that often focused on structured or supervised exercise programs [17,21]. Our study contributes new knowledge by illustrating that providers recognize a spectrum of activity, including light movement and daily tasks, as meaningful for cancer recovery, broadening the lens through which PA is integrated into survivorship care.

Interestingly, in the present study, oncology care providers mostly relied on verbal assessments to understand the patients’ prior or present experiences with PA. Assessing patients’ everyday activities in detail enhances PA evaluation by uncovering their mobility and sedentary behaviors, which helps to develop natural ways to include PA into their daily lives [14,39,52]. However, the lack of standardized tools across providers suggests variability in how PA is assessed and documented, which could affect care consistency and patient outcomes. Developing validated, brief screening tools may support more uniform assessment in busy oncology clinics.

Furthermore, some oncology care providers in the present study utilized the SPPB, a standardized tool used to assess lower extremity function and mobility, and followed NCCN guidelines, which provide evidence-based recommendations for cancer care, including physical activity promotion. This reflects a shift toward more standardized and objective assessments of PA in oncology settings. While physical activity is a critical component of cancer recovery, it remains a complex construct to measure, particularly when evaluating adherence to exercise prescriptions and their associations with health outcomes [57]. The adoption of objective measures, although not yet widespread, suggests growing interest in functional assessment tools that can inform tailored exercise prescriptions. Future research should examine the feasibility and acceptability of integrating such tools into routine care workflows.

In the present study, oncology care providers revealed an array of barriers that span knowledge gaps, infrastructural limitations, and environmental concerns. One major obstacle is the insufficient understanding of PA guidelines for individuals with cancer. Oncology care providers are unsure about the presence and details of these standards, which hinders their ability to provide consistent PA counseling. A pronounced deficiency in formal training in counseling cancer patients about physical activity compounds this issue. These results are consistent with prior studies showing that healthcare providers frequently lack sufficient training to recommend PA for cancer survivors, revealing a systemic deficiency in healthcare education [58,59,60]. Oncology care providers have acknowledged the need for more education and training on physical exercise to help cancer survivors. To achieve this, dedicated modules on PA counseling might be incorporated into medical and nursing curricula, and continuing education opportunities could be provided to current providers [39,61]. Incorporating PA competencies into provider certification and continuing medical education could also enhance accountability and sustainability of PA counseling practices.

Oncology care providers have noted additional barriers, such as the lack of prioritization of counseling and the feeling among providers that advising on PA falls outside their professional responsibilities [32,59,60]. These feelings indicate a wider cultural standard in oncology care that downplays the importance of lifestyle changes. Time constraints and limited resources hinder practitioners from offering thorough counseling, raising questions regarding the practicality of incorporating PA guidance into standard care practices [61,62]. Overcoming this cultural barrier may require institution-level policy changes and clinical leadership support to reframe PA as an essential component of cancer care, rather than an optional adjunct.

Oncology care providers have identified other barriers, including feeling unqualified to promote PA, insufficient community resources, and restricted referral options. These results align with those of previous studies conducted by Nadler et al. [32], Dilworth et al. [63], Hardcastle et al. [34], Schmitz et al. [39], and Mina et al. [64]. Implementing interdisciplinary care models with PA specialists might help prioritize PA counseling and clarify roles within cancer care teams, allowing all healthcare professionals to effectively address PA with patients [65,66,67]. Moreover, establishing referral systems for community resources or rehabilitation organizations could enhance the access to PA therapies [68,69,70]. Partnerships between oncology clinics and community-based fitness or rehabilitation programs may bridge this gap and ensure continuity of care beyond clinical settings.

Furthermore, cancer-related barriers, including poor health conditions and environmental factors such as living in risky areas or the threat of infection, make it harder for cancer survivors to engage in PA. Studies have regularly identified physical limitations, such as pain, fatigue, and medication side effects, as the main barriers. Clifford et al. [69] and Avancini et al. [70] have shown that physical side effects have a major impact on the activity levels of survivors. Additionally, barriers related to the environment, such as living in places that are thought to be dangerous or have an increased risk of illness, are also factors. This is consistent with the recent research conducted by Hirschey et al. [71], Jones & Paxton [72], and DeGuzman [73], who also recognize these difficulties. The convergence of these physical and environmental elements indicates a complex scenario of cancer survivors’ involvement in PA. This highlights the necessity for customized strategies that consider not only the physical capacities and restrictions of survivors but also the surroundings in which they reside [74,75]. To overcome these obstacles, strategies could involve tailored exercise regimens that consider individual side effects and conditions and community-wide initiatives focused on creating safer and more supportive environments for PA [57,66,76]. Mobile health (mHealth) interventions and home-based programs may also serve as scalable solutions to address environmental and transportation-related barriers.

In our study, oncology care providers recognized various facilitators that emerged as multidimensional approaches in oncology settings to promote PA among their patients. Acknowledging the health advantages linked to PA, such as enhanced bodily and mental health, serves as a primary motivator for providers, which acts as a basis for integrating PA counseling into standard oncology care practices [77]. Moreover, utilizing established PA standards from trustworthy sources provides a structured framework for clinical decision-making, offering a clear, evidence-based approach to efficiently recommend PA [78,79]. Following the PA guidelines, counseling is consistent with current research, maximizing patient health and recovery [80,81]. Furthermore, interdisciplinary support in PA counseling is crucial [82]. A coordinated approach with different professionals creates a comprehensive care strategy that meets the diverse requirements of cancer survivors [32,39]. Our findings suggest that when providers feel supported by institutional infrastructure and team-based workflows, they are more likely to integrate PA promotion into their clinical routines, supporting calls for systems-level solutions to sustain PA counseling in cancer care. This strategy emphasizes the need to consider all facets of a patient’s health, encompassing physical recuperation and emotional wellness, to enable a comprehensive approach to cancer care [81].

Overall, this study contributes new perspectives to the literature by showing how oncology care providers conceptualize PA more broadly, identify nuanced role- and setting-specific barriers, and navigate implementation through informal and formal strategies. These findings extend existing knowledge by offering context-specific evidence to support system-level and workforce-based improvements in PA integration within oncology care.

### Strengths and Limitations

To the best of our knowledge, this is one of the first studies to qualitatively explore oncology care providers’ perspectives on PA practices in their clinical settings in Nebraska. The strengths of the current study lie in the methods employed. The qualitative interview-based methodology enabled participants to express their opinions and experiences regarding specified issue areas. Additionally, the strength of the study lies in its systematic technique of gathering and analyzing data, which involves cross-referencing transcripts with audio recordings and field notes, as well as consensus-based triangulation among coders to ensure reliability and validity. Purposeful sampling was used to select a broad set of providers with different degrees of experience. Our research sets itself apart from previous studies [70,83] by including various healthcare professionals, including oncologists, nurses, and surgeons, who provide direct care and treatment to patients with cancer.

However, a key limitation is the study’s confinement to a single large hospital system in the Midwest region, which may restrict generalizability. Cultural norms, institutional policies, and workflow structures specific to this setting may not reflect those in other geographic or institutional contexts. Given the study’s focus on Nebraska, the findings may reflect region-specific healthcare practices shaped by both rural and urban care settings. Providers in rural areas may face unique challenges such as limited access to PA resources, transportation barriers, and fewer referral options, whereas urban providers may have greater infrastructure and programmatic support. Future studies across diverse geographic locations and healthcare systems are needed to validate and expand upon these findings. Additional studies with broader and more varied sample sizes are necessary to validate the applicability of these results. Moreover, there may be concerns regarding the self-selected sample, potentially resulting in the enrollment of oncology care providers who were motivated and enthusiastic about promoting PA to their patients. This could have impacted the conclusions derived from this study. The semi-structured interview questions were designed in a way that intended to ask questions openly and to reduce the likelihood of oncology care providers feeling obligated to address PA with patients. However, social desirability bias may have caused oncology care providers to alter their responses to appear favorable. To enhance trustworthiness and minimize interpretive bias, the study employed peer debriefing, triangulation, and reflexivity.

## 5. Conclusions

Oncology care providers demonstrate comprehensive awareness of PA, acknowledging its significance in everyday PA beyond the traditional engagement of activities. The study revealed barriers that hinder the successful counseling of PA for cancer survivors, including the lack of PA guidelines, insufficient training in PA counseling, and practical difficulties such as time constraints and limited resources. Despite these challenges, various factors facilitated PA counseling for cancer patients, such as the recognized health advantages of PA, understanding of current PA recommendations, availability of community PA resources, and support from interdisciplinary teams. Oncology care providers’ request for additional information on PA and a preference for receiving related training virtually demonstrate their willingness to address these obstacles. This study highlights the complex interplay between the barriers to and support for PA counseling within oncology care. These findings point to the need for improving provider education, simplifying referral procedures, and more prominently incorporating PA into cancer care protocols.

## Figures and Tables

**Table 1:** Socio-demographic characteristics of the oncology care providers (N = 16).

Characteristic	N (%)
Age (Years)	
19–39	5 (31.3)
40–49	7 (43.8)
50–59	2 (12.5)
60 and Above	2 (12.5)
Gender	
Male	4 (25)
Female	12 (75)
Race/Ethnicity	
White	12 (75)
Hispanic	2 (12.5)
Asian	2 (12.5)
Marital Status	
Single	2 (12.5)
Married	14 (87.5)
Clinical Specialty	
Staff Physician	8 (50)
Nurse Practitioner	2 (12.5)
Registered Nurse	2 (12.5)
Physician Assistant	2 (12.5)
Registered Dietician	1 (6.3)
Social Worker	1 (6.3)
Clinical Practice	
Medical Oncology	9 (52.2)
Surgical Oncology	2 (12.5)
Radiation Oncology	1 (6.3)
Symptom Management/Palliative care	2 (12.5)
Hematological Oncology	1 (6.3)
Gynecological Oncology	1 (6.3)
Cancer Treatment	
Breast Cancer	3 (18.8)
Hematological Cancer	3 (18.8)
Gynecological Cancer	1 (6.3)
Central nervous system Cancer	3 (18.8)
Mixed Cancer	6 (37.5)
Patient Type	
Outpatient	15 (93.3)
Inpatient	1 (6.3)
Years of Practice, Mean (SD)	11.75 (±10.35)

**Table 2:** Summary of themes and subthemes related to physical activity counseling within the oncology care context.

Theme	Subtheme	Example Quote
**Broad and Inclusive Conceptualizations of Physical Activity among Oncology Care Providers**	Defining physical activity	*“Being active in just about any capacity, whether it’s walking, aerobics, yoga.” [Registered Nurse]*
**Current Practices in Physical Activity Counseling**	Advice on physical activity	*“Usually, we start with small goals … to make them doable, manageable and seem achievable.” [Nurse Practitioner]*
	Assessment of physical activity	*“Well, when they’re first coming in for screening, we ask a lot of their social and previous history …” [Staff Physician]*
**Barriers to Physical Activity Counseling**	Lack of knowledge related to physical activity guidelines	*“I don’t know if there is a specific guideline.” [Staff Physician]*
	Lack of training related to physical activity counseling	*“Wasn’t ever touched [physical activity] upon with any population, really, but definitely not cancer patients.”*
	Low priority for physical activity counseling	*“I don’t ask specifically questions on their current physical levels of physical activity.” [Staff Physician]*
	Not being the right person to advise on physical activity	*“It’s not part of a checklist if you’re just the case manager.”*
	Time constraints	*“The physicians don’t seem to have time in their clinical role.”*
	Limited resources for physical activity counseling	*“Honestly, nothing. Because we don’t have any particular thing that we tell patients …” [Registered Nurse]*
	Limited referral opportunities	*“Our physicians, I’m so sorry. I don’t see our physicians referring them to PT or OT either.”*
	Inadequate health status of cancer survivors	*“And obviously, if it’s going to hurt them, if they’re already losing weight and not eating enough, I’m not going to tell them to increase physical activity.” [Staff Physician]*
	Environmental issues	*“I have patients that live in different parts of Omaha that don’t feel safe walking outside.”*
**Facilitators for Physical Activity Counseling**	Health benefits related to physical activity	*“Definitely mental health … I mean obviously also improves your circulation, lung health, cardiovascular.” [Staff Physician]*
	Knowledge of physical activity guidelines	*“150 min of moderate intensity physical activity a week for cancer survivors.” [Nurse Practitioner]*
	Resources available in the community	*“A clinical trial where they are looking at so they give patients a Fitbit, and they get health coaching.” [Staff Physician]*
	Interdisciplinary support	*“My case manager discusses [physical activity] with patient.” [Staff Physician]*
**Desire for Physical Activity Training and Education**	More education on physical activity	*“I guess the biggest thing is I want to know, if they’re able to do their, you know, activities of daily living and unable to function.” [Nurse Practitioner]*
	Training	*“I feel like it’s easier to have webinars or, you know, Zoom. Meeting conferences too are fine, but many people if it is more accessible if we do virtual.”*

## Data Availability

The de-identified qualitative data supporting the findings of this study are available from the corresponding author, Gaurav Kumar (gaurav-kumar-3@ouhsc.edu), upon reasonable request, and subject to Institutional Review Board restrictions on confidentiality.

## Supplementary Materials

The following supporting information can be downloaded at https://www.mdpi.com/article/10.3390/cancers17142281/s1: Document S1: Socio-demographic questionnaire for oncology care providers and Semi-structured questionnaire.
