# Supplementary material for: Perceived Barriers, Facilitators, and Needs Related to Promoting Physical Activity in Cancer Care: Qualitative Insights from Oncology Care Providers
Source: Cancers (Basel). 2025 Jul 9;17(14):2281. doi: 10.3390/cancers17142281 (PMC12293613; doi:10.3390/cancers17142281)
Supplement: Supplementary file 1 [file cancers-17-02281-s001.zip › cancers-3657347-supplementary.pdf]

S1: Socio-demographic questionnaire and semi-structured questionnaires

Socio-demographic questionnaire for Oncology care providers

1. **Age (Years)**

- ☐ 18-39
- ☐ 40-49
- ☐ 50-59
- ☐ 60- and more

2. **Gender**

- ☐ Male
- ☐ Female

3. **Race/Ethnicity**

- ☐ Non-Hispanic White
- ☐ Non-Hispanic Black
- ☐ Hispanic
- ☐ Others (Specify) ______________

4. **Marital status**

- ☐ Single
- ☐ Married/Living with a partner
- ☐ Divorced/Separated
- ☐ Widowed

5. **What regional cancer program are you primarily affiliated with?** Please specify_________________________________

6. **What is your primary clinical role?**

□ Staff physician

□ Registered nurse

□ Nurse practitioner

□ Radiation therapist

□ Other, please specify ____________________

7. What is your main area of clinical practice? (Select all that apply)

□ Surgical oncology

□ Radiation oncology

□ Medical oncology

□ Hematology/oncology

□ Symptom management & palliative care

□ Family practice

□ General medicine

□ Other, please specify_____________________

8. Please select the type of cancer(s) that is (are) your main area(s) of clinical focus:

□ General Practice

□ Breast Cancer

□ CNS Cancers

□ Gastrointestinal cancers

□ Genitourinary cancer

□ Gynecological cancer

□ Head & neck cancer

□ Hematological cancers (myeloma, lymphoma)

□ Hematological cancers (leukemia)

□ Lung cancer

□ Melanoma & skin cancers

□ Sarcoma

□ Other, please specify: _______________

9**. What is your main area of clinical practice (i.e., majority of your clinical time is spent in this area)?**

□ Inpatient

□ Outpatient

□ Both

10. How many years have you been in practice? __________

11. In your opinion, which professional(s) are most suited (i.e., have the knowledge, capabilities, and time) to discuss physical activity with patients? (Select all that apply)

□ Physicians

□ Nurses

□ Physiotherapists

□ Occupational therapists

□ Radiation therapists

□ Kinesiologists

□ Other, please specify ________

Semi-structured questions for the Oncology Care Providers

**Assess**	Beliefs and Knowledge:	• What do you think of when you hear the term “physical activity”?<br>• Do you know any of the benefits of physical activity for cancer survivors?<br>• Do you know any physical activity recommendations for cancer survivors? If yes, please elaborate.
	Abilities	• Are there any concerns or barriers you have regarding physical activity counseling?
	Assessment	• Do you discuss or advise cancer patients on physical activity? If No, then what are the factors that prevent you from counseling or recommendations: Are there any (professional, patient-related, or institutional barriers)?<br>• When do you recommend physical activity among cancer patients? During or after treatment?<br>• In what specific cancer type and during which stage, do you recommend your patients (cancer survivors) to perform physical activity?<br>• What questions do you ask to learn about cancer patients' physical activity levels?<br>• How do you assess the physical activity among cancer survivors? Please explain.
**Advise**	Dose of PA	• What types of physical activities do you typically prescribe?

		• How many minutes of physical activity would you recommend in a week?
	Health risks	• What barriers do you think cancer survivors have to be physically active?<br>• What do you think could help to overcome these barriers?
	Benefit of change	• How does physical activity help cancer survivors during and after cancer treatment? Please explain.
**Agree**	Motivation & Goals:	• Do you have any physical activity goals for cancer survivors? Please explain.<br>• Are there any factors that influence achieving the goal?
	Social Support	• Do you think any other member of your team discusses physical activity with their patients? Who?<br>• What social support do you think cancer survivors have for physical activity?
**Assist**	Environment Context and Resources:	• What resources are available for you to promote physical activity? Is there anything specific within their community that you would recommend?<br>• What aspects of the environment impact cancer survivors' physical activity?
	Referral	• Do you refer cancer survivors to a physical therapist, chiropractor, or kinesiologist?<br>• What are some facilitators and barriers when referring patients to other healthcare professionals?
**Arrange**	Specific Plan	• How do you monitor or follow up on the progress and maintenance of physical activity in cancer survivors?

		• Is it via visits, phone calls, or text messages? Please explain?

Additional questions

- Specific to physical activity, what type of information would you be interested in knowing? (Intensity, duration or type)
- Did you get any information regarding physical activity during your medical training/ certification?
- Do you need any education training related to physical activity promotion? Please explain.
- What type of training do you prefer (webinar, conference, educational materials, and resources)? Do you prefer virtual or in-person training? Any thoughts?
- If you were given an opportunity for physical activity recommendation to the cancer patients. What would it be? E.g.: education to the patient regarding PA (pamphlets, posters, handouts), increased motivation, enjoyable and settings goals in the PA program, cost accessible, targeted and flexible PA program.
